# Bis(dimethyl­ammonium) 3,3′-dicarb­oxy-5,5′-(5,7,12,14-tetra­oxo-6,13-diaza­tetra­cyclo­[6.6.2.0^4,16^.0^11,15^]hexa­deca-1,3,8,10,15-penta­ene-6,13-di­yl)dibenzoate dihydrate

**DOI:** 10.1107/S1600536812025470

**Published:** 2012-06-23

**Authors:** Lan-Ping Xu, Lan Qin, Lei Han

**Affiliations:** a State Key Laboratory Base of Novel Functional Materials and Preparation Science, Faculty of Materials Science & Chemical Engineering, Ningbo University, Ningbo, Zhejiang, 315211, People’s Republic of China; b State Key Laboratory of Structural, Chemistry, Fujian Institute of Research on the Structure of Matter, Chinese Academy of Sciences, Fuzhou, Fujian 350002, People’s Republic of China

## Abstract

The title compound, 2C_2_H_8_N^+^·C_30_H_12_N_2_O_12_^2−^·2H_2_O, comprises dimethyl­ammonium cations, 3,3′-dicarb­oxy-5,5′-(5,7,12,14-tetra­oxo-6,13-diaza­tetra­cyclo­[6.6.2.0^4,16^.0^11,15^]hexa­deca-1,3,8,10,15-penta­ene-6,13-di­yl)dibenzoate dianions and water mol­ecules. The dianion is situated on a crystallographic inversion centre. Two very strong symmetry-restricted O⋯H⋯O hydrogen bonds are present which are situated about the crystallographic inversion centres. In one of these hydrogen bonds, the H atom is situated at its centre, while in the other one the H atom is disordered about its centre. Both H atoms are involved in the chain-like *C* ^2^_2_(16) motif, and not in a more common motif *R* ^2^_2_(8) that is composed of a pair of hydrogen carboxyl­ates with the H atoms situated about the centre between the pair of O atoms. In the crystal, inter­action of these hydrogen bonds results in formation of anionic layers of dianions parallel to (-111). The water mol­ecules donate their H atoms to one of two of the carboxyl­ate O atoms, forming strong hydrogen bonds. The dimethyl­ammonium donates a bifurcated hydrogen bond to an oxo group of the dianion, forming weak hydrogen bonds. All the hydrogen bonds form a three-dimensional hydrogen-bonded network.

## Related literature

For organic supra­molecular solids, see: Pantos *et al.* (2007 ▶). For multi-component mol­ecular crystals or organic co-crystals, see: Bond (2007 ▶); MacGillivray (2008 ▶); Yan *et al.* (2011 ▶). For prediction of organic crystal structures, see: Pigge (2011 ▶). For organic structures based on naphthalaleneteracarb­oxy­lic diimide derivatives, see: Xu *et al.* (2011 ▶). For hydrogen carboxyl­ates forming chain-like motifs with very strong O—H⋯O hydrogen bonds, see: Foces-Foces *et al.* (1996 ▶); Hsu *et al.* (2006 ▶); Aciro *et al.* (2009 ▶). For *in situ* hydrolysis of dimethyl­formamide mol­ecules, see: Jain *et al.* (2008 ▶). For classification of hydrogen bonds, see: Desiraju & Steiner (1999 ▶). For graph-set motifs, see: Etter *et al.* (1990 ▶). For a description of the Cambridge Structural Database, see: Allen (2002 ▶). 
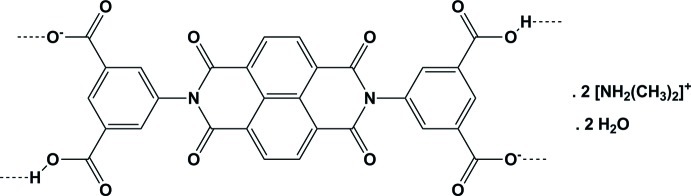


## Experimental

### 

#### Crystal data

2C_2_H_8_N^+^·C_30_H_12_N_2_O_12_^2−^·2H_2_O
*M* *_r_* = 720.64
Monoclinic, 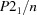

*a* = 10.428 (13) Å
*b* = 8.651 (10) Å
*c* = 18.40 (2) Å
β = 91.956 (16)°
*V* = 1659 (4) Å^3^
*Z* = 2
Mo *K*α radiation
μ = 0.11 mm^−1^
*T* = 293 K
0.20 × 0.20 × 0.20 mm

#### Data collection

Rigaku Saturn70 diffractometer
Absorption correction: multi-scan (*CrystalClear*; Rigaku/MSC, 2008 ▶) *T*_min_ = 0.788, *T*_max_ = 1.000
12286 measured reflections
3780 independent reflections
2137 reflections with *I* > 2σ(*I*)
*R*_int_ = 0.051

#### Refinement

*R*[*F* ^2^ > 2σ(*F* ^2^)] = 0.081
*wR*(*F* ^2^) = 0.266
*S* = 1.01
3780 reflections
248 parameters
3 restraints
H atoms treated by a mixture of independent and constrained refinement
Δρ_max_ = 0.43 e Å^−3^
Δρ_min_ = −0.36 e Å^−3^

### 

Data collection: *CrystalClear* (Rigaku/MSC, 2008 ▶); cell refinement: *CrystalClear*; data reduction: *CrystalClear*; program(s) used to solve structure: *SHELXS97* (Sheldrick, 2008 ▶); program(s) used to refine structure: *SHELXL97* (Sheldrick, 2008 ▶); molecular graphics: *SHELXTL* (Sheldrick, 2008 ▶); software used to prepare material for publication: *SHELXL97*.

## Supplementary Material

Crystal structure: contains datablock(s) I, global. DOI: 10.1107/S1600536812025470/fb2246sup1.cif

Structure factors: contains datablock(s) I. DOI: 10.1107/S1600536812025470/fb2246Isup2.hkl

Supplementary material file. DOI: 10.1107/S1600536812025470/fb2246Isup3.cml

Additional supplementary materials:  crystallographic information; 3D view; checkCIF report

## Figures and Tables

**Table 1:** Hydrogen-bond geometry (Å, °)

*D*—H⋯*A*	*D*—H	H⋯*A*	*D*⋯*A*	*D*—H⋯*A*
O1—H1⋯O1^i^	1.22 (1)	1.22 (1)	2.432 (5)	180 (1)
O4—H2⋯O4^ii^	1.15 (6)	1.45 (5)	2.441 (5)	139 (4)
N2—H2*B*⋯O5^iii^	0.90	2.22	2.850 (5)	127
O7—H3⋯O2^i^	0.98 (2)	1.95 (5)	2.805 (6)	144 (6)
O7—H4⋯O3^iv^	0.98 (2)	1.85 (4)	2.771 (6)	154 (7)

Symmetry codes: (i) 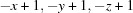; (ii) 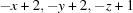; (iii) 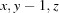; (iv) 
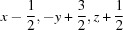
.

**Fig. 1.**
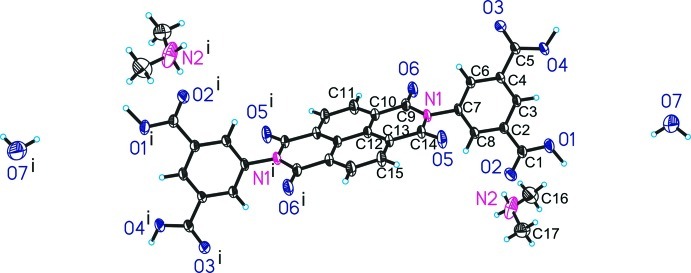
The title molecule with the displacement ellipsoids drawn at the 30% probability level and with the labelling scheme. The H atoms are shown as small spheres of arbitrary radii. (Symmetry code: i -x+1, -y+2, -z.)

**Fig. 2.**
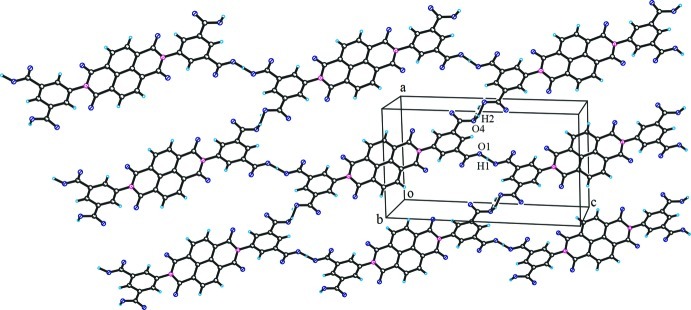
View of the anionic layer with very strong symmetry-restricted hydrogen bonds.

## supplementary crystallographic information

### Comment

Assemblies of functionalised organic molecules in the solid state have attracted much interest in crystal engineering and materials science (Pantos *et al.*, 2007). Recently, much attention has been paid to formation of multi-component molecular crystals or organic co-crystals as a means of modification of properties of organic molecules in the solid state (Bond, 2007; MacGillivray, 2008; Yan *et al.*, 2011). However, an effective strategy for tuning functionality of co-crystal solids still remains challenging (Pigge, 2011).

We have been interested in utilizing acid-functionalized naphthalaleneteracarboxylic diimide derivatives as starting materials in crystal engineering of a series of functional organic co-crystal materials (Xu *et al.*, 2011). Herein we report an organic salt, 2(C_2_H_8_N)^+^.(C_30_H_12_N_2_O_12_)^2-^.2H_2_O, which has been prepared under solvothermal reaction from 5,5-[naphthalene-1,8:4,5- bis(dicarboximide)-N,N-diyl]bis(benzene-1,3-dicarboxylic acid) and 1,10-phenanthroline in dimethylformamide (DMF). The dimethylammonium cations in the title structure were formed by *in situ* hydrolysis of the dimethylformamide molecules (Jain *et al.*, 2008).

Single-crystal X-ray diffraction analysis has indicated that the title structure is composed dimethylammonium cations, 3,3-dicarboxy-5,5-[naphthalene-1,8:4,5- bis(dicarboximide)-N,N-diyl]bis(benzene-1-carboxylate) anion and water molecules. As shown in Fig. 1, the anion is situated on the crystallographic inversion centre.

The most prominent as well as unusual feature of the title structure is presence of two different very strong symmetry restricted hydrogen bonds (Table 1; for the terminology of the hydrogen bonds, see Desiraju & Steiner, 1999). One of the hydrogens (H1) is situated at its centre while the other one (H4) is disordered about it as revealed the difference electron density maps. These hydrogens form a chain-like motif *C*^2^_2_(16) (Etter *et al.*, 1990). The atoms involved in this motif are as follows: H2···O4-C5-C4-C3-C2-C1-O1···H1···O1^i^-C1^i^-C2^i^-C3^i^-C4^i^-C5^i^-O4^i^···, where the symmetry code i = 1-*x*, 1-*y*, 1-*z*. This is only a fourth known example (Cambridge Structural Database (Allen, 2002; version 5.33)) of a chain motif in the hydrogen carboxylates with a strong or very strong hydrogen bond (up to 2.55Å for O···O) in contrast to 11 structures with a motif *R*^2^_2_(8) with the same type of the hydrogen bonds (up to 2.55Å for O···O) between the hydrogen carboxylates. The structures with the chain motif are as follows: (RABNEN, 4-(3,5-dimethylpyrazol-4-yl)benzoic acid trifluoroacetate, Foces-Foces *et al.* (1996); SERYUK, sesquikis(3,6-di(pyridin-4-yl)-1,2,4,5-tetrazine) trimesic acid dehydrate, Hsu *et al.* (2006); POYXOR, hemikis((1*RS*,2*RS*,3*RS*)-3-N,N-dibenzylaminocyclohexane -1,2-diol N-oxide) 3-chlorobenzoic acid, Aciro *et al.* (2009).

In the title structure, these short hydrogen bonds form 2D-layers (Fig. 2). The 2D-framework is extended to a 3D network by involvement of water which donates strong O—H···O hydrogen bonds to the oxo-groups of the hydrogen carboxylates. Dimethylammonium donates a weak bifurcated hydrogen bond to the oxo-group O5.

### Experimental

A mixture of 5,5-[naphthalene-1,8:4,5- bis(dicarboximide)-N,N-diyl]bis(benzene-1,3-dicarboxylic acid) (59.4 mg, 0.1 mmol), 1,10-phenanthroline (35.9 mg, 0.2 mmol) in dimethylformamide (3 ml) was sealed in a 25 ml teflon-lined stainless steel reactor and heated at 393 K for 72 h. Colourless and cube-like single crystals of the title compound were obtained after cooling to room temperature. The yield equals to 50 weight %.

### Refinement

All the hydrogens were discernible in the difference electron density maps. Notably in the final stages of the refinement it turned out that the hydrogens H1 and H2 involved in the symmetry restricted strong hydrogen bonds were situated just at the centre or disorded about it, respectively. The positional as well as the displacement parameters of these hydrogens have been refined. The positional parameters of the water hydrogens H3 and H4 were refined using the following restraints: The O7—H3 and O7—H4 distances equal to 0.965 (20) Å while for the angle was used restraint DANG 1.5555 (400) (*SHELXL97*; Sheldrick, 2008) which corresponds to the average angle H—Ow—H (107.407°) retrieved from the Cambridge Structural Database (CSD) (Allen, 2002) from the structures determined by neutron diffraction. The isotropic displacement parameters of these hydrogens (H3 and H4) were constrained as *U*_iso_(H) = 1.5*U*_eq_(O). Other H atoms were allowed to ride on their respective parent atoms at distances of C—H(phenyl) = 0.93 Å with *U*_iso_(H) = 1.2 *U*_eq_(C), C—H(methyl) = 0.96 Å with *U*_iso_(H) = 1.5*U*_eq_(C), N—H(ammonium) = 0.90 Å with *U*_iso_(H) = 1.2*U*_eq_(N).

### Crystal data

**Table:**

2C_2_H_8_N^+^·C_30_H_12_N_2_O_12_^2^^−^·2H_2_O	*F*(000) = 752
*M**_r_* = 720.64	*D*_x_ = 1.443 Mg m^−^^3^
Monoclinic, *P*2_1_/*n*	Mo *K*α radiation, λ = 0.71073 Å
Hall symbol: -P 2yn	Cell parameters from 3435 reflections
*a* = 10.428 (13) Å	θ = 2.2–27.6°
*b* = 8.651 (10) Å	µ = 0.11 mm^−^^1^
*c* = 18.40 (2) Å	*T* = 293 K
β = 91.956 (16)°	Cube-like, colourless
*V* = 1659 (4) Å^3^	0.20 × 0.20 × 0.20 mm
*Z* = 2	

### Data collection

**Table:**

Rigaku Saturn70 diffractometer	3780 independent reflections
Radiation source: fine-focus sealed tube	2137 reflections with *I* > 2σ(*I*)
Graphite monochromator	*R*_int_ = 0.051
Detector resolution: 28.5714 pixels mm^-1^	θ_max_ = 27.5°, θ_min_ = 3.2°
CCD_Profile_fitting scans	*h* = −13→13
Absorption correction: multi-scan (*CrystalClear*; Rigaku/MSC, 2008)	*k* = −11→11
*T*_min_ = 0.788, *T*_max_ = 1.000	*l* = −23→21
12286 measured reflections	

### Refinement

**Table:**

Refinement on *F*^2^	Primary atom site location: structure-invariant direct methods
Least-squares matrix: full	Secondary atom site location: difference Fourier map
*R*[*F*^2^ > 2σ(*F*^2^)] = 0.081	Hydrogen site location: difference Fourier map
*wR*(*F*^2^) = 0.266	H atoms treated by a mixture of independent and constrained refinement
*S* = 1.01	*w* = 1/[σ^2^(*F*_o_^2^) + (0.1621*P*)^2^] where *P* = (*F*_o_^2^ + 2*F*_c_^2^)/3
3780 reflections	(Δ/σ)_max_ < 0.001
248 parameters	Δρ_max_ = 0.43 e Å^−^^3^
3 restraints	Δρ_min_ = −0.36 e Å^−^^3^
0 constraints	

### Special details

**Table:**

Geometry. All esds (except the esd in the dihedral angle between two l.s. planes) are estimated using the full covariance matrix. The cell esds are taken into account individually in the estimation of esds in distances, angles and torsion angles; correlations between esds in cell parameters are only used when they are defined by crystal symmetry. An approximate (isotropic) treatment of cell esds is used for estimating esds involving l.s. planes.
Refinement. Refinement of F^2^ against ALL reflections. The weighted R-factor wR and goodness of fit S are based on F^2^, conventional R-factors R are based on F, with F set to zero for negative F^2^. The threshold expression of F^2^ > 2sigma(F^2^) is used only for calculating R-factors(gt) etc. and is not relevant to the choice of reflections for refinement. R-factors based on F^2^ are statistically about twice as large as those based on F, and R- factors based on ALL data will be even larger.

### Fractional atomic coordinates and isotropic or equivalent isotropic displacement parameters (Å^2^)

**Table:**

	*x*	*y*	*z*	*U*_iso_*/*U*_eq_	Occ. (<1)
N1	0.6155 (2)	0.9195 (3)	0.17822 (12)	0.0435 (6)	
C3	0.7165 (3)	0.7875 (3)	0.39335 (14)	0.0430 (6)	
H3A	0.7380	0.7588	0.4409	0.052*	
O6	0.7771 (2)	0.7754 (3)	0.13324 (12)	0.0697 (7)	
C6	0.7610 (3)	0.9351 (3)	0.28630 (15)	0.0473 (7)	
H6A	0.8123	1.0043	0.2617	0.057*	
C12	0.5244 (2)	0.9859 (3)	0.03593 (13)	0.0402 (6)	
C7	0.6535 (3)	0.8733 (3)	0.25224 (13)	0.0415 (6)	
C8	0.5761 (3)	0.7673 (3)	0.28718 (14)	0.0425 (6)	
H8A	0.5040	0.7259	0.2633	0.051*	
O5	0.4661 (3)	1.0808 (3)	0.22384 (12)	0.0720 (8)	
O3	0.9850 (2)	1.0402 (3)	0.36519 (13)	0.0745 (8)	
C14	0.5140 (3)	1.0235 (3)	0.17045 (14)	0.0459 (7)	
C4	0.7929 (3)	0.8936 (3)	0.35776 (14)	0.0442 (7)	
C13	0.4669 (3)	1.0575 (3)	0.09499 (14)	0.0444 (7)	
O1	0.5588 (3)	0.5959 (3)	0.46612 (13)	0.0822 (9)	
H1	0.5000	0.5000	0.5000	0.17 (4)*	
C2	0.6085 (3)	0.7243 (3)	0.35853 (14)	0.0409 (6)	
C9	0.6833 (3)	0.8543 (4)	0.12086 (15)	0.0473 (7)	
C1	0.5278 (3)	0.6114 (4)	0.39882 (16)	0.0538 (8)	
C10	0.6314 (3)	0.8857 (3)	0.04610 (14)	0.0452 (7)	
C15	0.3628 (3)	1.1555 (4)	0.08394 (16)	0.0569 (8)	
H15A	0.3255	1.2033	0.1232	0.068*	
C5	0.9058 (3)	0.9650 (4)	0.39769 (17)	0.0555 (8)	
C11	0.6858 (3)	0.8183 (4)	−0.01297 (16)	0.0590 (8)	
H11A	0.7565	0.7538	−0.0058	0.071*	
N2	0.3624 (5)	0.1938 (5)	0.3549 (2)	0.1119 (15)	
H2A	0.4089	0.2749	0.3401	0.134*	
H2B	0.3412	0.1375	0.3152	0.134*	
C16	0.2451 (4)	0.2531 (6)	0.3843 (3)	0.0923 (13)	
H16A	0.1929	0.2981	0.3459	0.138*	
H16B	0.2656	0.3303	0.4203	0.138*	
H16C	0.1990	0.1701	0.4062	0.138*	
C17	0.4449 (5)	0.0987 (6)	0.4027 (3)	0.1022 (15)	
H17A	0.5121	0.0541	0.3752	0.153*	
H17B	0.3950	0.0178	0.4235	0.153*	
H17C	0.4819	0.1619	0.4409	0.153*	
O2	0.4408 (3)	0.5385 (3)	0.36690 (14)	0.0844 (10)	
O4	0.9083 (2)	0.9425 (3)	0.46707 (12)	0.0759 (8)	
H2	0.965 (5)	1.036 (6)	0.500 (3)	0.040 (16)*	0.50
O7	0.6740 (4)	0.5344 (6)	0.7689 (2)	0.1276 (14)	
H3	0.620 (6)	0.552 (9)	0.725 (2)	0.191*	
H4	0.611 (6)	0.540 (9)	0.808 (3)	0.191*	

### Atomic displacement parameters (Å^2^)

**Table:**

	*U*^11^	*U*^22^	*U*^33^	*U*^12^	*U*^13^	*U*^23^
N1	0.0534 (13)	0.0541 (13)	0.0224 (11)	−0.0007 (10)	−0.0079 (9)	0.0064 (9)
C3	0.0529 (15)	0.0514 (15)	0.0242 (13)	−0.0022 (12)	−0.0059 (11)	0.0043 (11)
O6	0.0693 (14)	0.1023 (18)	0.0368 (13)	0.0242 (13)	−0.0068 (10)	0.0118 (12)
C6	0.0531 (15)	0.0600 (17)	0.0282 (14)	−0.0161 (13)	−0.0071 (11)	0.0086 (12)
C12	0.0476 (14)	0.0478 (14)	0.0249 (14)	−0.0043 (12)	−0.0036 (11)	0.0055 (10)
C7	0.0525 (14)	0.0500 (15)	0.0214 (13)	−0.0042 (12)	−0.0082 (11)	0.0021 (10)
C8	0.0495 (14)	0.0488 (15)	0.0285 (14)	−0.0065 (12)	−0.0082 (11)	0.0037 (11)
O5	0.0876 (17)	0.1009 (19)	0.0271 (12)	0.0284 (14)	−0.0023 (11)	−0.0034 (11)
O3	0.0663 (14)	0.111 (2)	0.0455 (14)	−0.0401 (14)	−0.0119 (11)	0.0173 (13)
C14	0.0555 (16)	0.0581 (16)	0.0238 (14)	−0.0009 (13)	−0.0035 (11)	0.0045 (11)
C4	0.0467 (14)	0.0576 (16)	0.0277 (14)	−0.0103 (12)	−0.0087 (11)	0.0040 (11)
C13	0.0514 (15)	0.0588 (16)	0.0226 (13)	−0.0014 (13)	−0.0049 (11)	0.0043 (11)
O1	0.1009 (19)	0.107 (2)	0.0378 (13)	−0.0553 (16)	−0.0163 (13)	0.0252 (13)
C2	0.0485 (14)	0.0443 (14)	0.0294 (14)	−0.0070 (11)	−0.0032 (11)	0.0035 (11)
C9	0.0530 (15)	0.0601 (17)	0.0286 (14)	0.0031 (14)	−0.0032 (12)	0.0076 (12)
C1	0.0645 (18)	0.0637 (18)	0.0327 (16)	−0.0190 (15)	−0.0064 (13)	0.0127 (13)
C10	0.0502 (15)	0.0582 (16)	0.0269 (14)	−0.0009 (13)	−0.0047 (11)	0.0084 (11)
C15	0.0705 (19)	0.074 (2)	0.0262 (14)	0.0165 (16)	−0.0010 (13)	−0.0003 (13)
C5	0.0581 (18)	0.075 (2)	0.0327 (16)	−0.0185 (16)	−0.0135 (13)	0.0072 (14)
C11	0.0644 (18)	0.079 (2)	0.0332 (16)	0.0206 (16)	−0.0042 (13)	0.0068 (14)
N2	0.180 (4)	0.084 (2)	0.075 (3)	0.040 (3)	0.051 (3)	0.013 (2)
C16	0.083 (3)	0.096 (3)	0.098 (4)	−0.009 (3)	0.002 (2)	0.004 (2)
C17	0.099 (3)	0.109 (3)	0.098 (4)	0.019 (3)	−0.007 (3)	−0.019 (3)
O2	0.0893 (18)	0.106 (2)	0.0561 (16)	−0.0564 (16)	−0.0266 (13)	0.0311 (14)
O4	0.0778 (16)	0.115 (2)	0.0328 (12)	−0.0436 (15)	−0.0197 (11)	0.0107 (12)
O7	0.128 (3)	0.165 (4)	0.090 (3)	−0.030 (3)	0.017 (2)	−0.026 (3)

### Geometric parameters (Å, º)

**Table:**

N1—C14	1.393 (4)	C2—C1	1.501 (4)
N1—C9	1.408 (4)	C9—C10	1.486 (4)
N1—C7	1.461 (3)	C1—O2	1.237 (4)
C3—C2	1.389 (4)	C10—C11	1.373 (4)
C3—C4	1.394 (4)	C15—C11^i^	1.403 (4)
C3—H3A	0.9300	C15—H15A	0.9300
O6—C9	1.208 (4)	C5—O4	1.291 (4)
C6—C7	1.374 (4)	C11—C15^i^	1.403 (4)
C6—C4	1.393 (4)	C11—H11A	0.9300
C6—H6A	0.9300	N2—C16	1.448 (6)
C12—C13	1.403 (4)	N2—C17	1.463 (6)
C12—C10	1.421 (4)	N2—H2A	0.9000
C12—C12^i^	1.421 (5)	N2—H2B	0.9000
C7—C8	1.393 (4)	C16—H16A	0.9600
C8—C2	1.395 (4)	C16—H16B	0.9600
C8—H8A	0.9300	C16—H16C	0.9600
O5—C14	1.222 (4)	C17—H17A	0.9600
O3—C5	1.224 (4)	C17—H17B	0.9600
C14—C13	1.486 (4)	C17—H17C	0.9600
C4—C5	1.499 (4)	O4—H2	1.15 (6)
C13—C15	1.387 (4)	O7—H3	0.98 (2)
O1—C1	1.276 (4)	O7—H4	0.98 (2)
O1—H1	1.216 (2)		
			
C14—N1—C9	125.5 (2)	O2—C1—C2	120.8 (3)
C14—N1—C7	117.0 (2)	O1—C1—C2	114.8 (2)
C9—N1—C7	117.5 (2)	C11—C10—C12	119.9 (2)
C2—C3—C4	120.7 (2)	C11—C10—C9	120.5 (3)
C2—C3—H3A	119.7	C12—C10—C9	119.5 (3)
C4—C3—H3A	119.7	C13—C15—C11^i^	119.4 (3)
C7—C6—C4	119.7 (3)	C13—C15—H15A	120.3
C7—C6—H6A	120.2	C11^i^—C15—H15A	120.3
C4—C6—H6A	120.2	O3—C5—O4	124.9 (3)
C13—C12—C10	121.5 (2)	O3—C5—C4	120.8 (3)
C13—C12—C12^i^	119.8 (3)	O4—C5—C4	114.3 (3)
C10—C12—C12^i^	118.8 (3)	C10—C11—C15^i^	121.5 (3)
C6—C7—C8	121.5 (2)	C10—C11—H11A	119.3
C6—C7—N1	120.6 (2)	C15^i^—C11—H11A	119.3
C8—C7—N1	117.9 (2)	C16—N2—C17	117.5 (4)
C7—C8—C2	119.0 (2)	C16—N2—H2A	107.9
C7—C8—H8A	120.5	C17—N2—H2A	107.9
C2—C8—H8A	120.5	C16—N2—H2B	107.9
O5—C14—N1	120.6 (3)	C17—N2—H2B	107.9
O5—C14—C13	122.7 (3)	H2A—N2—H2B	107.2
N1—C14—C13	116.7 (2)	N2—C16—H16A	109.5
C6—C4—C3	119.4 (2)	N2—C16—H16B	109.5
C6—C4—C5	121.0 (3)	H16A—C16—H16B	109.5
C3—C4—C5	119.6 (2)	N2—C16—H16C	109.5
C15—C13—C12	120.6 (3)	H16A—C16—H16C	109.5
C15—C13—C14	119.3 (3)	H16B—C16—H16C	109.5
C12—C13—C14	120.0 (3)	N2—C17—H17A	109.5
C1—O1—H1	117.0 (2)	N2—C17—H17B	109.5
C3—C2—C8	119.7 (2)	H17A—C17—H17B	109.5
C3—C2—C1	119.2 (2)	N2—C17—H17C	109.5
C8—C2—C1	121.1 (2)	H17A—C17—H17C	109.5
O6—C9—N1	120.6 (3)	H17B—C17—H17C	109.5
O6—C9—C10	123.0 (3)	C5—O4—H2	114 (3)
N1—C9—C10	116.4 (2)	H3—O7—H4	102 (4)
O2—C1—O1	124.4 (3)		

Symmetry code: (i) −*x*+1, −*y*+2, −*z*.

### Hydrogen-bond geometry (Å, º)

**Table:**

*D*—H···*A*	*D*—H	H···*A*	*D*···*A*	*D*—H···*A*
O1—H1···O1^ii^	1.22 (1)	1.22 (1)	2.432 (5)	180 (1)
O4—H2···O4^iii^	1.15 (6)	1.45 (5)	2.441 (5)	139 (4)
N2—H2*B*···O5^iv^	0.90	2.22	2.850 (5)	127
O7—H3···O2^ii^	0.98 (2)	1.95 (5)	2.805 (6)	144 (6)
O7—H4···O3^v^	0.98 (2)	1.85 (4)	2.771 (6)	154 (7)

Symmetry codes: (ii) −*x*+1, −*y*+1, −*z*+1; (iii) −*x*+2, −*y*+2, −*z*+1; (iv) *x*, *y*−1, *z*; (v) *x*−1/2, −*y*+3/2, *z*+1/2.
